# Obstructive sleep apnea mouth breathing phenotype response to combination oral appliance therapy

**DOI:** 10.3389/frsle.2024.1272726

**Published:** 2024-03-06

**Authors:** Preetam Schramm, Emet Schneiderman, Jason Hui, Zohre German, William Stenberg, Ju Ying Lin

**Affiliations:** Department of Biomedical Sciences, Texas A&M University College of Dentistry, Dallas, TX, United States

**Keywords:** oral appliance, mouth shield, sleep apnea, mouth breathing, phenotype, snoring

## Abstract

**Introduction:**

Obstructive sleep apnea (OSA) is a multisystem physiological disorder of breathing during sleep that may contribute to systemic physiological imbalances and can also be exacerbated by the use of some commonly prescribed medications.

**Methods:**

In a randomized parallel design trial, we included phenotypic mild to severe OSA mouth-breathing subjects (*n* = 36) confirmed by home polygraphy, to evaluate the efficacy of oral appliance plus mouth shield and oral appliance only during sleep on night 1 (T1) after 4 weeks (T2), and after 8 weeks (T3) of oral appliance therapy. Respiratory dynamics data were collected. Primary outcomes were respiratory event index and mouth breathing. Anamnesis on medication intake was collected at enrollment.

**Results:**

The respiratory event index and the hypopnea index did not statistically differ between groups at T3. Oral appliance plus mouth shield and oral appliance only significantly reduced mouth breathing at T2 (*p* = 0.012) and T3 (*p* ≤ 0.001) compared with baseline. Exploratory analyses showed oral appliance plus mouth shield supine respiratory rate at T3 (*p* = 0.039) was marginally decreased compared with oral appliance only. The snore percentage did not differ statistically between groups at T3. Oral appliance only showed a marginal oxygen saturation increase (*p* = 0.019) at T3 compared with oral appliance plus mouth shield. At T3, medication users had persistent respiratory events, mouth breathing, and snoring compared with non-medication users. Logistic regression showed medication use may increase the odds of mouth breathing (OR = 1.148; *p* = 0.015) and snoring (OR = 1.036; *p* = 0.049).

**Discussion:**

In our OSA-mouth breathing cohort, oral appliance only was similar to oral appliance plus mouth shield in attenuating the respiratory event index, hypopnea index, and mouth breathing after 8 weeks. Oral appliance only increased oxygen saturation at T3, while oral appliance plus mouth shield maintained a relatively narrow oxygen saturation range from T1–3. Oral appliance plus mouth shield marginally lowered the supine respiratory rate at T3 compared with oral appliance only. Persistent respiratory events, mouth breathing, and snoring were observed in medication users at T3.

## Introduction

One billion people throughout the world are estimated to have obstructive sleep apnea (OSA) (Lyons et al., 2020). A physiological feature in the pathogenesis of OSA is collapsibility of the upper airway and snoring. The area of the tongue base (oropharynx) and soft palate (nasopharynx) are musculomembranous airflow-restrictive sites that show greater airflow resistance and are believed to be more susceptible to collapse during sleep (Zhao et al., 2013; Chen et al., 2018). The size of the nasopharynx can determine whether the mode of breathing is nasal or oral, with mouth breathing resulting in poorer sleep quality (Grewal and Godhane, 2010). Furthermore, at sleep onset, centrally mediated reduction in respiratory drive occurs and the upper airway undergoes both functional and structural changes. These changes lead to spatially and temporally distributed sites particularly at the oropharynx in OSA patients (Chen et al., 2018) and are conducive to snore sound generation (Abeyratne et al., 2013). Snoring is considered the prodromal phase of OSA and can lead to partial upper airway collapse (hypopnea) or complete upper airway blockage (apnea) and associated oxygen desaturations. In healthy volunteers who snored, a higher percentage of mouth breathing during sleep occurred compared with those who did not snore (Gleeson et al., 1986). Mouth-breathing children were more likely to snore and have clinically significant apnea–hypopnea indexes (AHI) than nose-breathing children (Juliano et al., 2009). Mouth-breathing patients with moderate-to-severe respiratory disturbance indexes (sum of # of apneas and hypopneas/h and # of respiratory event-related arousals/h during sleep) were less adherent to continuous positive airway pressure (CPAP) therapy to treat their OSA and a high percentage of study participants continued to mouth breathe with CPAP compared with nose-breathers (Bachour and Maasilta, 2004). A recent study involving 21 OSA participants used an adhesive mouthpiece tape (AMT) to keep the mouth closed during sleep. AMT combined with various mandibular advancement devices (MAD + AMT) or oral appliance (OA + AMT) designs resulted in 52% (11/21) of subjects having persistent AHI ≥ 15 events/h, but the combined therapy lowered the median AHI significantly compared with MAD or AMT alone. However, mouth breathing response to MAD + AMT was not reported and the study's exclusion criteria did not list possible study omissions with current medication use (Labarca et al., 2022). An information gap persists regarding whether combination oral appliance interventions, their impact on respiratory dynamics, and the response to treatment to attenuate mouth breathing are efficacious. Furthermore, paired oral appliance therapy in conjunction with medication use requires elucidation of a respiratory event index, mouth breathing, and snoring response.

Oral appliance therapy functions to create greater oropharyngeal space by advancing the mandible forward (Walsh et al., 2008). It decreases airway collapsibility to enable proper breathing compared with no oral appliance intervention. Oral appliances are also recommended as the first-line treatment for mild to moderate OSA and second-line therapy for severe OSA (Trzepizur et al., 2021) or in those who refuse CPAP (ADA Delegates, 2017). Mouth shields have been used to address malocclusions (Tallgreen et al., 1998) and as comfort devices with oral appliances (Schramm et al., 2021). Buccal soft tissues, the tongue, and teeth comprise a barrier unit between atmospheric extra-oral environmental and intra-oral functional space that even maintains a specific pressure when closed (Moss and Salentijn, 1969). Maintenance of an optimal intra-oral pressure facilitates proper tongue position and mouth closure (Knösel et al., 2010). Moreover, preventing mouth breathing and facilitating nasal breathing creates ventilation efficiency while reducing snoring (Rappai et al., 2003; Edwards and White, 2011), respiratory resistance (Meurice et al., 1996), apneas and hypopneas (Bachour and Maasilta, 2004), airway collapsibility (Morais-Almeida et al., 2019), and blood oxygen saturation decreases (Fleury et al., 2004), along with oral hygiene improvements (Keris et al., 2016).

In this study, we paired a midline traction oral appliance with a mouth shield positioned in the oral vestibule to attenuate mouth breathing. Oral appliance plus mouth shield allowed us to explore interrelated hypotheses about respiratory dynamics occurring on night one and after 4 and 8 weeks. The first hypothesis was that oral appliance plus mouth shield would demonstrate superiority in reducing the respiratory event index and mouth breathing compared with oral appliance only. Secondary hypotheses were that oral appliance plus mouth shield would lower (1) respiratory rate, (2) attenuate snoring, and (3) improve oxygen saturation response during sleep compared with oral appliance only. Exploratory analyses were performed to determine whether prescribed medications influenced persistent respiratory event index, mouth breathing, and snoring.

## Materials and methods

### Study design

**Figure 1:**
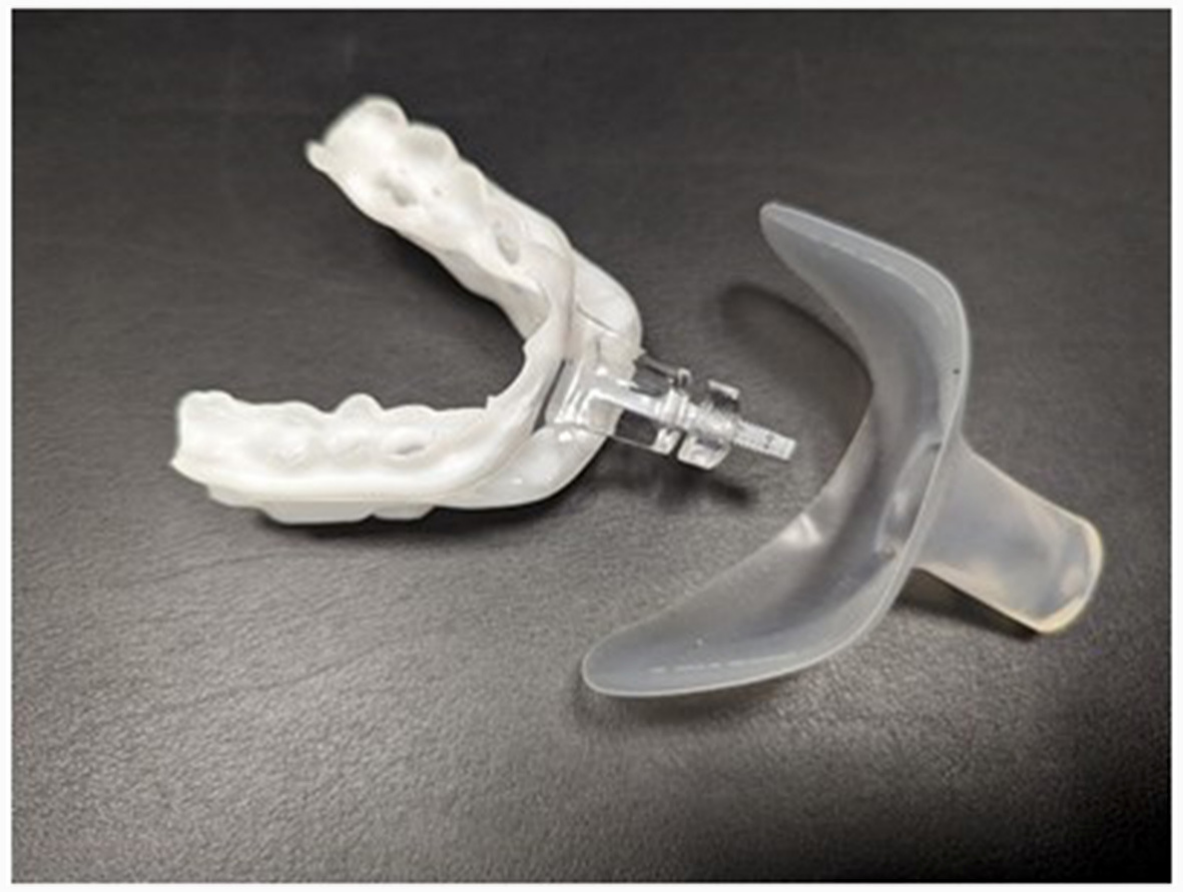
myTAP oral appliance plus mouth shield.

The parallel study design was a prospective randomized control trial using oral appliance plus mouth shield (myTAP^®^ plus mouth shield; AMI Inc. Carrollton, TX) and the same oral appliance without the mouth shield (Figure 1).

All eligible participants were consecutively enrolled and randomly assigned to either oral appliance plus mouth shield or oral appliance only. Subjects were included based on home sleep test confirmation of mouth breathing, snoring, and a respiratory event index of >5 events/h and no history of oral appliance use. Additional inclusion criteria included unobstructed nasal breathing (breathing through the nose for 1–2 min with mouth closed), which was assessed while awake by the team's dentist at the time of the oral exam; a minimum of eight stable teeth per arch to support the oral appliance; healthy gums, jaw joints, and muscles. Subjects were excluded in the absence of mouth breathing, a respiratory event index of <5 events/h, obstructed nasal breathing, and not meeting oral health conditions to support the oral appliance. We did not exclude participants using prescribed medications or actively recruited medication-using subjects (Supplementary Table 1). The randomization sequence was generated using Online Research Randomizer software, Version 4.0. Written informed consent was obtained from all participants. The study was approved by the Institutional Review Board at Texas A&M University, College of Dentistry (IRB2019-0421-CD-FB) and registered at ClinicalTrials.gov Identifier: NCT04876625. https://classic.clinicaltrials.gov.

### Oral appliance

Subjects were instructed to use their oral appliance plus mouth shield or oral appliance only nightly. Oral appliance plus mouth shield and oral appliance only were started at ~60% of the subject's maximum protrusion position. All subjects received oral and written instructions to adjust the oral appliance in compliance with the manufacturer's recommendations. After acclimatizing to the oral appliance's initial position, subjects were instructed to advance their mandibles up to two turns (0.25 mm each) per night if snoring, if OSA events or daytime sleepiness persisted, or if they did not experience discomfort. T1 and T2 sleep recording results were used as a guide for instructing subjects to make titration adjustments.

### Home sleep test

Home sleep recordings were collected at baseline to confirm the presence of mouth breathing and OSA events and establish their severity. Two consecutive night recordings were attempted at T1, the start of oral appliance plus mouth shield and oral appliance only use; at T2, 4 weeks from the start; and at T3, 8 weeks from the start. Data from T1–3 recordings when available were used as a single observation to capture night-to-night variability. Respiratory dynamics data including respiratory event index were obtained and analyzed using the NOX T3 recorder and software (NOX Medical, Reykjavík, Iceland). Peripheral oxygen saturation percentage [% range: 0–100%; High resolution one (1) second sampling rate] was measured during the recording period with a Nonin finger probe pulse oximeter (Model 3150, Nonin Medical Inc. MN, USA). Each subject received instructions on the recorder's operation and how to self-apply sensors. A minimum of five recorded hours without artifacts and recording all channels was considered acceptable. Study and oral appliance compliance was defined as completing the study protocol from T1–3 using the assigned intervention of >5 h/night self-reported in the subject's sleep diary upon awakening. Apnea and hypopnea events were visually scored using the revised American Academy of Sleep Medicine 2007 scoring criteria (Berry et al., 2012). The respiratory event index was defined as the sum of all apneas and hypopnea events/hour (apneas, >90% reduction in airflow from baseline, hypopneas, 30–90% airflow reduction from baseline associated with ≥3% oxygen desaturation and duration ≥10 s). The hypopnea index (HI, #events/h) used similar hypopnea event criteria. Respiratory rate (#breaths/minute), oxygen desaturation index (ODI, #events/h with ≥3% oxygen desaturation), and oxygen saturation (%), mouth breathing (#minutes; ≥3 breaths minimum duration ≥20 dB), and snore percentage (snore minutes ≥ 20 dB/analysis duration minutes) were obtained. Snoring and mouth breathing sounds were manually scored if they were in synchrony with breathing effort and protuberant from the background noise using the NOX T3 built-in audio sensor (Arnadottir et al., 2015). Differentiation between nasal snoring without mouth breathing vs. snoring with mouth breathing relied on snore pattern recognition in the Audio and Audio Volume (dB) signals. Nasal snoring presents with a crescendo-decrescendo “diamond shape” type pattern in the Audio signal and a plateau peak in the Audio Volume (dB) signal. Both of these signal characteristics are absent during mouth breathing. The recorder placement followed the manufacturer's recommended mid-thoracic montage.

### Statistical analysis

R Studio Statistical package (version 1.4.1103) and SPSS v20 software (IBM Inc., Chicago) were used for computations. Most of the sleep study variables were not normally distributed, so frequencies, medians, and interquartile ranges (IQR) were used for description. Fisher's exact test was used to determine possible differences between the two treatment groups in ancestry. Bonferroni-corrected Mann-Whitney *U*-tests were used for identifying significant between-group differences for continuous variables. An adjusted alpha level of 0.0125 was used for each of these pairwise tests; *p*-values between 0.0125 and 0.05 were characterized as marginally significant. Spearman's rho correlation coefficient and prevalence ratio were used to assess between-group associations. Logistic regression models were used to analyze associations between non-users and medication users with mouth breathing and snoring as outcome variables. These tests used an alpha significance level of *p* < 0.05.

## Results

### Subjects

**Figure 2:**
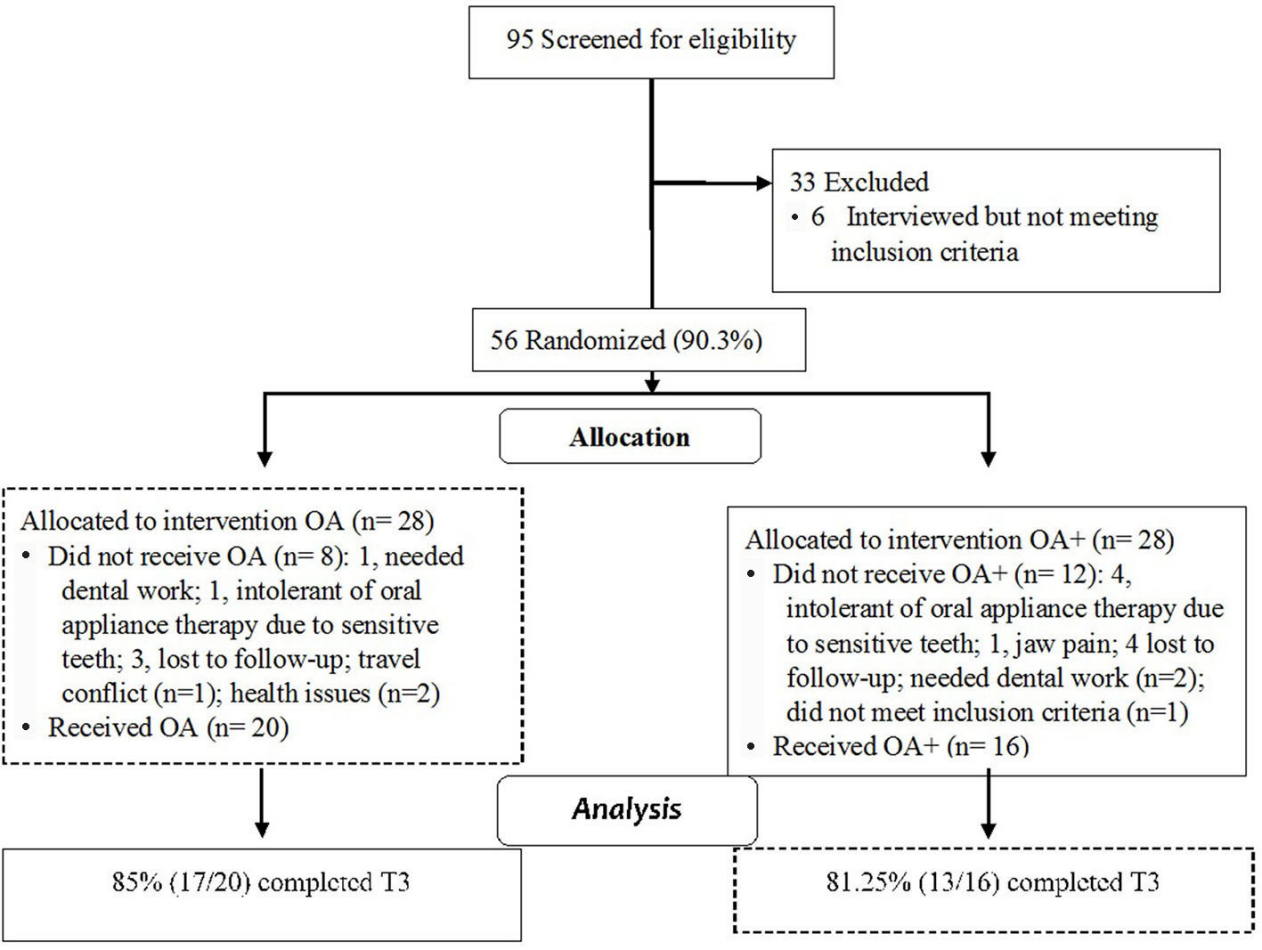
Cohort flow diagram with attrition; OA+, oral appliance plus mouth shield; OA, oral appliance.

A total of 95 potential subjects comprised the screened cohort, and 62 (65%) received an interview, 56 (90.3%) of whom were enrolled in the study. In total, 20 subjects dropped out due to travel conflicts (1), health issues (2), needing dental work (3), being uncomfortable with OA during oral fitting (5), jaw pain (1), lost to follow-up (7), and not meeting respiratory event index inclusion criteria (1) (Figure 2). A total of 16 and 20 subjects in the oral appliance plus mouth shield and oral appliance-only groups, respectively, were included. The distribution of baseline characteristics by group assignment, oral appliance plus mouth shield vs. oral appliance only, is provided in Table 1. No statistical difference in sex distribution between the oral appliance plus mouth shield and the oral appliance-only groups was observed (56.3 vs. 70% male, respectively; Chi-square = 0.728, *p* = 0.393). Overall, subjects self-reported their ancestries as follows: 25 European/white (69.4%), 2 African American (5.6%), 2 Hispanic (5.6%), 3 American Indian (8.3%), and 4 Asian (11.1%). None of the observed differences in ancestry groups according to treatment were significant (European/white: 62.5 vs. 75%; African American: 12.5 vs. 0%; Hispanic: 0 vs. 10%; American Indian: 12.5 vs. 5%; Asian 12.5 vs. 10%; Fisher's exact test, *p* = 0.354). There were no significant group differences with regard to BMI, respiratory event index, hypopnea index, respiration rate, snore count, and oxygen saturation. The oxygen saturation was marginally higher in the oral appliance plus mouth shield group at baseline (*p* = 0.044).

**Table 1:** Subject characteristics by group assignment at baseline.

**Variable**	**Oral appliance plus mouth shield**	**Oral appliance**	***P*-value**
	**Median (IQR) (*****N*** = **16)**	**Median (IQR) (*****N*** = **20)**	**Between groups**
Age (years)	61.5 (56–68.25)	62 (55.7–70.0)	0.213
BMI	26.7 (25.5–32)	27.6 (27.1–28.4)	0.281
Sex [# (%) male]	9 (56.3)	14 (70.0)	0.393
Ancestry [#; (%)]			0.354
European	10 (62.5)	15 (75.0)	0.557
African American	2 (12.5)	0	0.500
Hispanic	0	2 (10.0)	0.500
American Indian	2 (12.5)	1 (5.0)	1.00
Asian	2 (12.5)	2 (10.0)	1.00
**Respiration**			
Respiratory event index (# events/h)	21.5 (11.3–43.6)	18.4 (13.0–34.1)	0.824
Hypopnea index (# events/h)	15.0 (8.8–18.8)	14.8 (9.9–19.6)	0.588
Oxygen desaturation index (ODI)	20.5 (12.4–31.2)	23.1 (13.9–32.1)	0.494
Oxygen saturation (%)	93.5 (92.4–94.2)	91.7 (90.4–92.5)	0.044
Snore count (#)	169.6 (68.1–294.7)	190.8 (38.0–241.0)	0.445
Respiratory rate (breaths/m)	16.9 (13.6–19.1)	16.3 (14.5–19.7)	0.762
Respiratory rate non-supine (breaths/m)	16.9 (14.6–18.3)	15.5 (14.5–19.5)	0.842
Respiratory rate supine (breaths/m)	16.9 (13.5–21.8)	16.0 (13.8–19.6)	0.644
**Dentition**			
Over-jet (mm) (±SD)	2.53 (±1.85)	2.43 (±1.23)	0.863
Over-bite % (±SD)	35 (±31.05)	40 (±24.64)	0.628

IQR, interquartile range (25th−75th percentile).

Of the attempted sleep recordings, the oral appliance plus mouth shield group had 16 (100%) at T1, 15 (93.7%) at T2, and 13 (81.2%) at T3. The number of sleep recordings collected in the oral appliance-only group was 20 (100%) at T1, 20 (100%) at T2, and 17 (85%) at T3. The majority of rejected recordings were due to technical issues (i.e., lost oxygen saturation signal) and <5 h of recorded time. At T3, three subjects in the oral appliance plus mouth shield group (18.75%) and three subjects in the oral appliance-only group (15%) had incomplete sleep recordings. No major adverse events related to the use and titration of oral appliance plus mouth shield or oral appliance only were reported. At T3, compliance based on self-reporting with oral appliance plus mouth shield was 81.25% (13/16) and 85% (17/20) with oral appliance only.

### Dental assessment

All subjects had >8 teeth per arch. The oral appliance plus mouth shield group vs. the oral appliance-only group median overjet and over-bite percentage did not differ between groups. Crowding in the upper and lower dentitions was in 8.1 and 16.2% of the subjects, respectively. Anterior cross-bite was observed in 5.4% and posterior cross-bite in 5.4% of subjects. Temporomandibular joints were within normal limits in 100% of subjects. The overall mean maximum mouth opening was 50.1 mm. Mandibular protrusion produced by 60% of maximal mandible protrusion at baseline was 4.5 mm (3.6–5.5) in the oral appliance plus mouth shield group and 4.5 mm (2.7–6.2) in the oral appliance-only group. None of these variables differed significantly between groups (*p* > 0.05).

### Respiratory event index and hypopnea index

At baseline and T1–3, no statistical differences in the respiratory event index were observed between groups. No statistical differences were found between the groups' respiratory event index reduction from baseline (Table 2).

**Table 2:** Respiratory event index and hypopnea index: oral appliance plus mouth shield and oral appliance only response at T1–3.

**Variable**	**Oral appliance plus mouth shield**	**Oral appliance only**	***P*-value**
**Respiratory event index (#events/h)**	**Median (IQR) (*****N*** = **16)**	**Median (IQR) (*****N*** = **20)**	**Between groups**
Baseline (*n* = 36)	21.5 (11.3–43.6)	18.4 (13.0–34.1)	0.824
T1	9.5 (4.65–15.20)	5.3 (2.87–9.37)	0.080
T2	6.9 (3.85–14.9)	5.8 (2.42–8.70)	0.355
T3	6.9 (4.0–16.0)	6.8 (2.6–9.32)	0.335
**REI** Δ **from baseline**			
T1	−8.1 (−30.7 to −5.1)	−9.8 (−30.9 to −5.2)	0.378
T2	−12.7 (−28.5 to −4.9)	−12.2 (−25.9 to −7.9)	0.247
T3	−9.5 (−27.7 to −3.6)	−13.6 (−28.41 to −7.9)	0.425
**Hypopnea index (HI; #events/h)**			
Baseline (*n* = 36)	15.0 (8.8–18.8)	14.8 (9.9–19.6)	0.588
T1	7.8 (3.4–9.0)	5.9 (3.2–9.0)	0.655
T2	6.4 (3.7–14.1)	4.4 (2.6–8.7)	0.133
T3	8.3 (3.6–11.8)	5.1 (3.1–7.6)	0.643
**HI** Δ **from baseline**			
T1	−4.7 (−6.3 to 0.5)	−7.6 (−18.6 to −2.2)	0.087
T2	−3.9 (−7.6 to −2.2)	−7.7 (−15.1 to −3.8)	0.042
T3	−5.5 (−8.3 to −1.5)	−9.4 (−14.9 to −4.4)	0.054

REI, respiratory event index (#events/hour); T1, night 1; T2, after 4 weeks; T3, after 8 weeks of intervention; IQR, interquartile range (25th−75th percentile); Δ from baseline, change from baseline.

Logistic regression analyses were performed to assess the probability of medication intake as a risk factor for increased respiratory event index at T3. After controlling for age and sex, medication intake did not increase the odds of respiratory events (increase in apneas plus hypopneas/hour) [odds = 1.06, 95% CI (0.97–1.16), *p* = 0.151].

The HI at baseline and T1–3 was not statistically different between groups. At T2, the HI reduction from baseline was marginally greater (*p* = 0.042) with oral appliance only, but no statistical differences (*p* = 0.054) between groups at T3 were observed (Table 2).

Logistic regression analyses were performed to assess the probability of medication intake as a risk factor for increased hypopnea index at T3. After controlling for age and sex, medication intake was observed to increase the odds of hypopnea [odds = 1.13, 95% CI (1.00–1.26), *p* = 0.038].

### Mouth breathing

**Figure 3:**
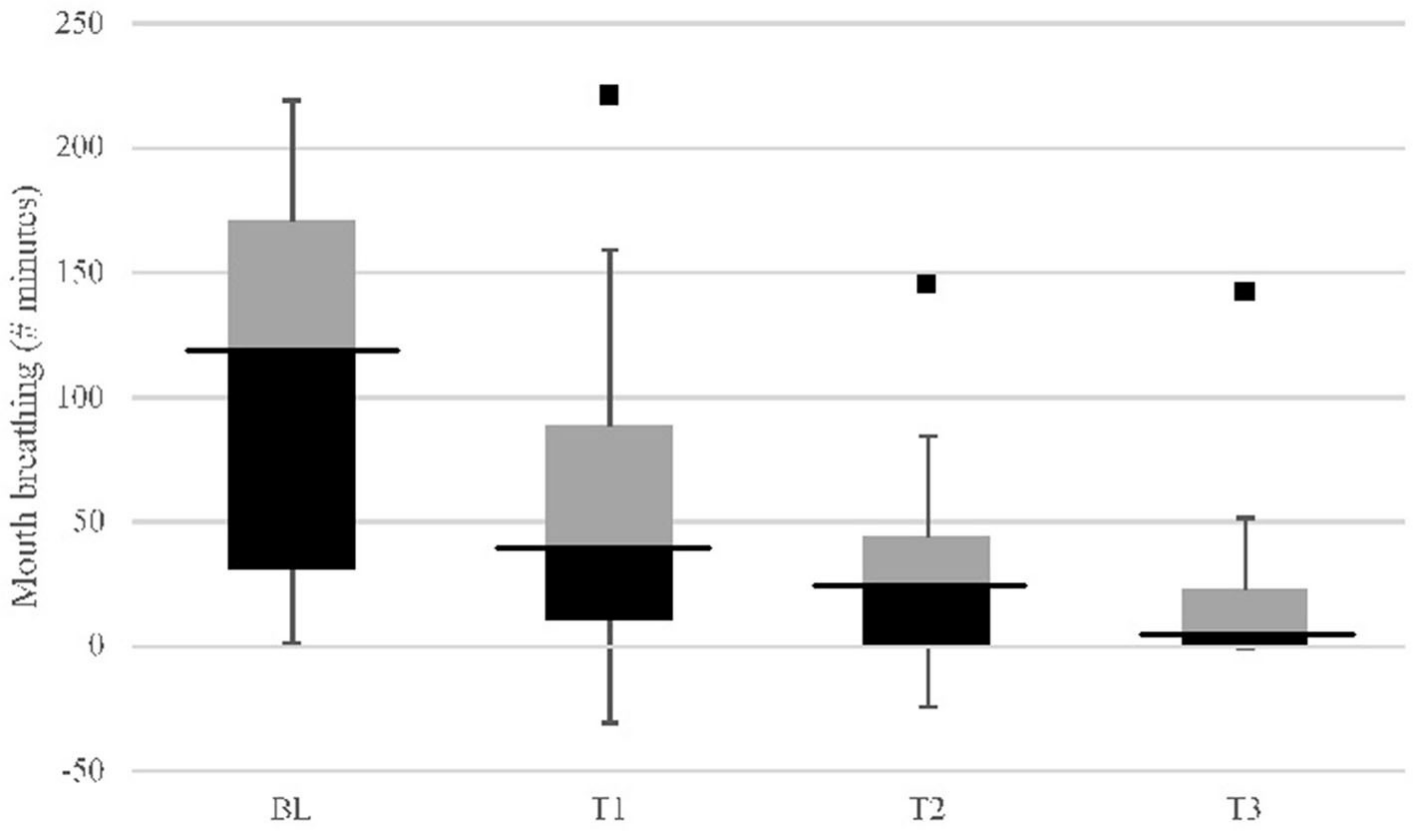
Mouth breathing (minutes) study group (*n* = 30) response comparison: baseline (BL) vs. 8-weeks (T3) with OA+ and OA (IQR). Comparison of baseline (BL) mouth breathing to: *p* < 0.01; T2, *p* < 0.001; T3, *p* < 0.001. Comparison of T1 to: T2, *p* = 0.012; T3, *p* < 0.001. Comparison of T2 to: T3, *p* = 0.148 [without outlier (black square point) using Tamsulosin, *p* = 0.011]; Inter quartile range (IQR) column: black, 25th–50th percentile; black bar, median; gray, 50th–75th percentile.

Mouth breathing minutes among all participants (*n* = 30) at T1 (*p* < 0.01), T2 (*p* < 0.001), and T3 (*p* < 0.001) were significantly reduced compared with baseline (Figure 3). Mouth breathing minutes at T2 (*p* = 0.012) and T3 (*p* < 0.001) were significantly reduced compared with T1. Without an outlier (Tamsulosin user), T3 mouth breathing was significantly lower (*p* = 0.011) compared with T2. Within-group comparison showed oral appliance plus mouth shield (*p* = 0.001) and oral appliance only (*p* < 0.001) significantly reduced mouth breathing at T3 compared with baseline. At T3, persistent mouth breathing was observed in 7/13 (53.8%) subjects with oral appliance plus mouth shield after titration and in 10/17 (58.8%) subjects with oral appliance only. No between-group differences were observed in subjects who did and did not mouth breathe. All (100%; *n* = 17) subjects with persistent mouth breathing used prescribed medication(s) (Supplementary Table 1). The remaining six (6) (46.2%) subjects in the oral appliance plus mouth shield group and seven (7) (41.2%) subjects in the oral appliance-only group were not medication users and did not mouth breathe at T3.

Mouth breathing comparison between non-users and medication users at baseline did not differ significantly. At T3, mouth breathing was significantly lower (*p* = 0.007) in non-users compared with medication users. The percentage reduction in mouth breathing at T3 was significantly greater (*p* = 0.001) in the non-users compared with medication users (Supplementary Table 4).

Logistic regression analyses were performed to assess the probability of medication intake as a risk factor for mouth breathing at T3. After controlling for age and sex, medication intake was observed to increase the odds of mouth breathing percentage reduction (i.e., persistent mouth breathing) (odds = 1.148, 95% CI = 1.027–1.284; *p* = 0.015).

### Respiratory rate (breaths/min)

At T2, the change in respiratory rate from baseline was marginally lower (*p* = 0.033) in the oral appliance plus mouth shield group compared with the oral appliance-only group. No statistical differences (*p* = 0.437) were observed at T3 between groups (Table 3).

**Table 3:** Respiratory rate: oral appliance plus mouth shield and oral appliance only response at T1–3.

**Variable**	**Oral appliance plus mouth shield**	**Oral appliance only**	***P*-value**
**RR total**	**Median (IQR) (*****N*** = **16)**	**Median (IQR) (*****N*** = **20)**	**Between groups**
Baseline (*n* = 36)	16.9 (13.6–19.1)	16.3 (14.5–19.7)	0.762
T1	16.8 (14.5–18.7)	15.9 (15.2–22.2)	0.814
T2	16.0 (13.6–18.0)	17.9 (14.5–21.2)	0.080
T3	16.4 (14.5–17.6)	16.6 (14.0–18.6)	0.729
**RR** Δ **from baseline**			
T1	−0.1 (−0.7 to 1.3)	0.3 (−0.12 to 1.9)	0.258
T2	−0.9 (−2.6 to 0.9)	0.8 (−0.1 to 1.2)	0.033
T3	−0.4 (−2.6 to 1.0)	0.1 (−2.5 to 0.7)	0.439

RR, respiratory rate (breaths/minute); T1, night 1; T2, after 4 weeks; T3, after 8 weeks of intervention; IQR, inter-quartile range (25th−75th percentile); Δ from baseline, change from baseline.

Respiratory rate non-supine at T2 was marginally slower (*p* = 0.045) with oral appliance plus mouth shield compared with oral appliance only. No statistical differences (*p* = 0.487) were observed at T3 between groups. Respiratory rate non-supine change from the baseline was marginally decreased (*p* = 0.044) with oral appliance plus mouth shield at T2 compared with oral appliance only. No statistical differences (*p* = 0.637) were observed at T3 between the groups (Table 4).

**Table 4:** Respiratory rate (non-supine and supine): oral appliance plus mouth shield and oral appliance only response at T1–3.

**Variables**	**Oral appliance plus mouth shield**	**Oral appliance only**	**Between groups**
**Respiratory rate non-supine (breaths/min)**	**Median (IQR) (*****N*** = **16)**	**Median (IQR) (*****N*** = **20)**	***P*** **-value**
Baseline (*n* = 36)	16.9 (14.6–18.3)	15.5 (14.5–19.5)	0.842
T1	17.5 (14.5–18.7)	16.4 (15.2–22.2)	0.594
T2	16.6 (13.6–18.0)	16.6 (14.5–21.2)	0.045
T3	16.1 (14.0–17.6)	16.1 (14.8–18.6)	0.487
**RR** Δ **from baseline**			
T1	0.1 (−0.7 to 1.3)	1.0 (−0.12 to 1.9)	0.104
T2	−0.1 (−2.6 to 0.9)	0.5 (−0.1 to 1.2)	0.044
T3	−0.6 (−2.6 to 1.0)	0.1 (−2.5 to 0.7)	0.637
**Respiratory rate supine (breaths/min)**			
Baseline (*n* = 36)	16.9 (13.5–21.8)	16.0 (13.8–19.6)	0.644
T1	16.3 (15.2–19.3)	17.2 (15.2–18.6)	0.151
T2	16.9 (13.0–20.6)	17.9 (15.2–20.5)	0.066
T3	16.7 (13.4–19.9)	17.3 (15.0–18.4)	0.487
Δ **from baseline**			
T1	0.3 (−1.8 to 0.8)	1.1 (−0.4 to 2.1)	0.051
T2	−0.3 (−2.8 to 0.9)	1.5 (0.5 to 3.1)	0.020
T3	−0.7 (−3.3 to 0.5)	0.1 (−1.0 to 2.1)	0.039

RR, respiratory rate (breaths/minute); T1, night 1; T2, after 4 weeks; T3, after 8 weeks of intervention; IQR, interquartile range (25th−75th percentile); Δ from baseline, change from baseline.

The respiratory rate supine did not differ statistically between the oral appliance plus mouth shield and oral appliance-only groups at baseline, T1, and T3. At T2, the respiratory rate supine change from baseline was marginally lower (*p* = 0.020) in the oral appliance plus mouth shield group and at T3 (*p* = 0.039) compared with the oral appliance-only group (Table 4).

### Snore percentage (%)

Snore percentage did not differ statistically between the oral appliance plus mouth shield and oral appliance-only groups at baseline, T2, and T3. At T1, the snore percentage was marginally higher (*p* = 0.027) in the oral appliance plus mouth shield group compared with the oral appliance-only group. The snore percentage change from baseline was not significantly different between groups at T1–3 (Table 5).

**Table 5:** Snore percentage (%) of analysis duration (minutes): oral appliance plus mouth shield and oral appliance only response at baseline, T1–3.

**Variable**	**Oral appliance plus mouth shield**	**Oral appliance only**	**Between groups**
**Snore percentage (%)**	**Median (IQR) (*****N*** = **16)**	**Median (IQR) (*****N*** = **20)**	***P*** **-value**
Baseline (*n* = 36)	23.3 (8.4–32.6)	22.3 (6.4–32.8)	0.924
T1	8.8 (2.8–24.4)	5.8 (1.6–16.9)	0.027
T2	6.0 (1.2–16.6)	10.0 (1.9–26.2)	0.611
T3	7.8 (0.2–29.0)	5.5 (1.1–20.4)	0.876
**Snore percentage** Δ **from baseline**			
T1	−11.1 (−24.9 to 7.4)	−9.6 (−32.4 to 3.6)	0.174
T2	−10.9 (−23.5 to 2.4)	−12.9 (−23.8 to −0.2)	0.506
T3	−10.9 (−23.4 to 2.8)	−4.8 (−24.8 to 10.0)	0.404

T1, night 1; T2, after 4 weeks; T3, after 8 weeks of intervention; IQR, inter-quartile range (25th−75th percentile); Δ from baseline, change from baseline.

Logistic regression analyses were performed to assess the probability of medication intake as a risk factor for increased persistent snoring at T3. After controlling for age and sex, medication intake was not observed at T3 (odds = 1.02, 95% CI = 0.99–1.05, *p* = 0.068) to increase the odds of snore percentage (i.e., persistent snoring).

### Oxygen saturation and oxygen desaturation index

The peripheral oxygen saturation at baseline was marginally higher (*p* = 0.044) in the oral appliance plus mouth shield compared with the oral appliance-only group. T1–3 oxygen saturation did not differ between groups. At T1 (*p* = 0.003), T2 (*p* = 0.017), and T3 (*p* = 0.019), oxygen saturation change from baseline was significantly less with oral appliance plus mouth shield compared with oral appliance only (Table 6).

**Table 6:** Oxygen saturation and oxygen desaturation index (ODI): oral appliance plus mouth shield and oral appliance only response at T1–3.

**Variables**	**Oral appliance plus mouth shield**	**Oral appliance only**	**Between groups**
**Oxygen saturation (%)**	**Median (IQR) (*****N*** = **16)**	**Median (IQR) (*****N*** = **20)**	***P*** **-value**
Baseline (*n* = 36)	93.5 (92.4–94.2)	91.7 (90.4–92.5)	0.044
T1	92.8 (92.1–94.0)	93.7 (92.3–95.1)	0.198
T2	93.6 (93.0–94.5)	93.5 (93.3–94.9)	0.875
T3	93.6 (92.4–94.2)	93.8 (93.2–94.9)	0.685
Δ **from baseline**			
T1	−0.6 (−0.8 to 0.3)	2.5 (0.5 to 3.7)	0.003
T2	0.5 (−0.3 to 0.6)	3.5 (0.9 to 3.5)	0.017
T3	−0.3 (−0.8 to 0.3)	2.9 (−1.7 to −0.1)	0.019
**ODI (#events/h)**			
Baseline (*n* = 36)	18.1 (9.0–33.1)	20.6 (9.6–26.9)	0.494
T1	7.1 (5.0–14.3)	7.0 (4.9–12.3)	0.270
T2	8.0 (4.1–11.9)	9.3 (5.9–15.3)	0.671
T3	7.1 (4.3–12.5)	10.6 (3.2–14.5)	0.540
Δ **from baseline**			
T1	−7.6 (−23.7 to −0.4)	−6.6 (−20.9 to −2.3)	0.245
T2	−9.0 (−21.1 to −0.9)	−8.1 (−19.1 to −1.1)	0.659
T3	−8.6 (−21.0 to −2.5)	−8.5 (−17.4 to −3.9)	0.762

T1, night 1; T2, after 4 weeks; T3, after 8 weeks of intervention; IQR, inter-quartile range (25th−75th percentile); Δ from baseline, change from baseline; ODI, oxygen desaturation index (#events/hour).

The ODI at baseline and T1–3 and change from baseline at T1–3 did not differ statistically between groups (Table 6).

## Discussion

Studies using custom-fitted oral appliances (Byun et al., 2020; Schneiderman et al., 2021; Labarca et al., 2022) or temporary oral appliances (Schramm et al., 2021; Segù et al., 2021) confirm significant AHI or respiratory event index and snoring reduction, regardless of oral appliance brand or design. However, these investigations included heterogeneous OSA patients, making the interpretation of oral appliance efficacy among the OSA mouth breathing phenotype difficult. We report on the performance of oral appliance plus mouth shield in phenotypic OSA mouth-breathing subjects to reduce the respiratory event index, mouth breathing, and snoring compared with the performance of oral appliance only. Our hypothesis was that oral appliance plus mouth shield would show superiority in improving respiratory dynamics during sleep compared with oral appliance only.

Contrary to our first hypothesis, we found oral appliance plus mouth shield and oral appliance only were similarly effective in reducing the respiratory event index on night 1 and after 4 and 8 weeks in mild to severe OSA subjects who self-titrated their custom, dentist-fitted oral appliance. Our novel approach demonstrated that mouth breathing attenuation with oral appliance plus mouth shield or oral appliance only did not differ after 8 weeks. The supine respiratory rate change from baseline at T2–3 was marginally decreased with oral appliance plus mouth shield. These results complement the report by Edwards and colleagues, who found that oral appliances improved passive anatomical collapsibility and ventilation (Edwards et al., 2016). Enabling nasal breathing using the added mouth shield likely improved ventilation efficiency (Teschler et al., 1999), while both oral appliance plus mouth shield and oral appliance only reduced snoring (Rappai et al., 2003; Norrhem and Marklund, 2016; Schramm et al., 2021) and upper airway resistance (Meurice et al., 1996). Our data suggest that oral appliance plus mouth shield use likely restored some processes of homeostatic neuromuscular control, attenuated collapsibility, and improved chemo-reflex dynamics (i.e., lower loop gain resulting in a lower oxygen saturation percentage change from baseline at T1–3 and a decrease in supine respiratory rate) compared with the use of oral appliance only. Oral appliance only increased oxygen saturation from baseline at T1–3. Exploratory investigation within each group showed that persistent mouth breathing and snoring were present in those using at least one prescribed medication vs. non-medication users. Furthermore, at T3, non-users had a lower respiratory event index (6.4 events/h) compared with medication users (17.8 events/h) (Supplementary Table 2). The results of non-users vs. medication users are presented in the Appendix.

In brief, our exploratory investigation found that medication use increased the risk of persistent respiratory events, hypopneas, and mouth breathing at T3 with either oral appliance plus mouth shield or oral appliance only. This supports the work of others who found antidepressant use increased the odds of sleep bruxism 2-fold (de Baat et al., 2021; Massahud et al., 2022), a sleep disorder associated with OSA events (Saito et al., 2013). Our novel finding could be relevant to healthcare professionals treating OSA with oral appliance therapy. Recognition that a relationship exists between medication use, persistent mouth breathing, and hypopneas in oral appliance-treated patients could facilitate communication among healthcare providers to develop a multidisciplinary treatment approach to optimize upper airway patency. Medication usage and phenotyping subjects based on low loop gain (Marklund et al., 2019) and mouth breathing may provide important tools regarding subject selection processes for clinical research protocols investigating oral appliance therapy efficacy.

Similar to the overall respiratory event index results, the hypopnea index at T1–3 did not differ between the oral appliance plus mouth shield and oral appliance-only group. Oral appliance plus mouth shield and oral appliance-only group responses suggest advancement attenuated predominant apneas and support the results of a recent study on oral appliance efficacy (Byun et al., 2020). The authors suggest that anatomical changes produced by oral appliance advancement might affect static obstructions (apneas) more than dynamic (hypopneas) obstructions, thus explaining the increased proportion of hypopnea episodes they observed in their study population after 1 month of oral appliance treatment. In contrast to their increased hypopnea results, we observed decreased hypopneas with both oral appliance plus mouth shield and oral appliance only. Furthermore, their study methods description does not provide any information on medication use in the study population. Our data show that the hypopnea index reduction was not statistically different between groups at T3 or when non-users and medication users were compared; however, the median HI was <5 events/h in non-users compared with medication users (6.0 events/h) (Supplementary Table 3).

Sleep onset is associated with reduced upper airway tone, which predisposes to sleep apnea (Chua and Heneghan, 2006), and in turn, fragments sleep through increased afferent feedback from multiple structures including the upper airway, respiratory effort mechanisms, and peripheral chemo-reflex (Mansukhani et al., 2014). Arousals from sleep cause blood pressure surges (Carrington and Trinder, 2008) and increase heart function from concomitant increases in sympatho-excitatory activity responses. Measuring respiratory effort changes in response to increased or decreased airway resistance should not only reflect pathological states (Boas et al., 2013; Edwards et al., 2016) but also identify effective therapy (Schramm et al., 2021).

Oral appliance plus mouth shield marginally decreased the supine respiratory rate at T2 and at T3 compared with oral appliance only. This finding supports a growing body of evidence indicating that the normal entry of air into the nostrils synchronizes slow respiration (Fontanini and Bower, 2006). The mouth shield enabled normal airflow through the nose that likely resulted in the entrainment of delta and theta rhythms and the synchronization of cellular networks, including limbic systems that can modify the rate and depth of breathing (Ito et al., 2014; Zelano et al., 2016; Heck et al., 2017). Our study supports the finding that respiratory rate is lower in nasal-breathing children than in mouth-breathing children (Boas et al., 2013). While the small decrease observed in supine respiratory rate with oral appliance plus mouth shield may not have clinical significance, taken together with the percentage reduction in the respiratory event index, snoring and mouth breathing in non-medication users after 8 weeks and consistent oxygen saturation stability among both non-medication and medication users within the oral appliance plus mouth shield group demonstrates the mouth shield's importance in restoring some measures of physiological homeostasis. Future studies could withdraw the mouth shield to determine whether respiratory dynamics increase to further evaluate the treatment response.

Our study results on snoring with oral appliance plus mouth shield is another novel finding that adds to the growing body of evidence regarding oral appliance-associated benefits. Although snore percentage was not statistically different between groups at baseline, T1–3, our data support the report from Norrhem and Marklund (2016) who used an oral appliance with elastic bands to control mouth opening during sleep. They observed a lower, non-statistically different snoring time percentage in subjects using oral appliance with elastic bands compared with oral appliance only. However, when our study population was divided into non-users and medication users, the snore percentage showed a 2-fold reduction at T3 among non-users (Supplementary Table 5). Preventing mouth breathing and facilitating nasal breathing with a mouth shield likely increased ventilation efficiency, while both interventions reduced snoring, airway collapsibility, respiratory resistance, apneas, and hypopneas (Meurice et al., 1996; Rappai et al., 2003; Bachour and Maasilta, 2004; Fleury et al., 2004; Morais-Almeida et al., 2019). Persistent snoring at T3 was likely influenced by the commingling of non-users with medication users within each treatment group. The data are presented in the Supplementary Table 5.

In contrast to past reports that found no oxygen saturation (Labarca et al., 2022) or minimum oxygen desaturation index (Norrhem and Marklund, 2016) differences between groups, our study observed oxygen saturation response differences between groups. The baseline oxygen saturation was marginally higher in the oral appliance plus mouth shield group and oxygen saturation changes at T1–3 oscillated within a narrow range (−0.6 to −0.05%) compared with the oral appliance-only group oxygen saturation that ranged from 2.5 to 3.5% and increased at T3 from baseline. The oxygen saturation stability observed with oral appliance plus mouth shield from T1–3 supports other findings showing that nasal-breathing children maintained higher oxygen saturation values compared to mouthbreathing children (Juliano et al., 2009; Boas et al., 2013). This finding suggests that the mouth shield likely facilitated oxygen saturation chemo-reflex stability, keeping the oxygen saturation average close to baseline levels. The T1 acute oxygen saturation improved with oral appliance only and maintained at T2–3, suggesting that this group's response was likely attributable to mandible advancement and supports the findings of the study by Segù et al. (2021), which used a similar temporary oral appliance to our study. We found no medication influence on oxygen saturation variables.

High treatment adherence and oral appliance preference are often reported in studies comparing oral appliance and CPAP (Trzepizur et al., 2021). This study had protocol completion and oral appliance use compliance based on participants' self-reporting of 81.25% (13/16; oral appliance plus mouth shield) and 85.0% (17/20; oral appliance only).

We believe that despite the study's limitations, including its small sample size, multiple comparisons, and conducting the study during the COVID-19 lockdown, which limited our patient access and end-of-treatment evaluations, it contributes to disentangling some of the complexities associated with OSA and effective therapies. The strength of this study is in its parallel design with data collection at three time points in evaluating the efficacy of oral appliance plus mouth shield. We also demonstrate the influence of some medications on respiratory dynamics and airway management. Another important limitation was assigning sleep recorders to subjects outside the facility during COVID-19 lockdowns, which may have contributed to the technical recording issues encountered. COVID-19 conditions contributed to lower study participation at T3 and our inability to obtain end-of-study oral appliance advancement measures.

## Conclusion

To our knowledge, this is the first study to utilize the combination therapy oral appliance plus mouth shield in OSA mouth-breathing patients. Both interventions reduced the respiratory event index after 8 weeks in non-users of medication. We demonstrate that oral appliance plus mouth shield lowers supine respiratory rate and both interventions attenuate mouth breathing. Oral appliance plus mouth shield may also support chemo-reflex stability relative to the observed small oxygen saturation range changes and oral appliance only increased oxygen saturation. Some medications may induce persistent respiratory events, snoring, and mouth breathing with either oral appliance therapy.

## Data availability statement

The datasets presented in this article are not readily available because participant consent form states their data will not be shared. Requests to access the datasets should be directed to ES, eschneiderman@tamu.edu.

## Ethics statement

The studies involving humans were approved by Review Board at Texas A&M University, College of Dentistry IRB2019-0421-CD-FB. The studies were conducted in accordance with the local legislation and institutional requirements. The participants provided their written informed consent to participate in this study.

## Author contributions

PS: Conceptualization, Formal analysis, Methodology, Writing – original draft, Validation. ES: Funding acquisition, Methodology, Project administration, Supervision, Writing – review & editing, Validation. JH: Investigation, Project administration, Supervision, Writing – review & editing. ZG: Data curation, Validation, Writing – review & editing. WS: Investigation, Project administration, Writing – review & editing. JL: Investigation, Methodology, Project administration, Writing – review & editing.
