# Supplementary material for: Obstructive sleep apnea mouth breathing phenotype response to combination oral appliance therapy
Source: Front Sleep. 2024 Mar 6;3:1272726. doi: 10.3389/frsle.2024.1272726 (PMC12713827; doi:10.3389/frsle.2024.1272726)
Supplement: Supplementary file 1 [file Data_Sheet_1.docx]

# **Appendix A**

The following section describes observations on respiratory dynamics in sleep that includes: respiratory event index, hypopnea index, mouth breathing and Snore percent in non-medicated (non-users) and medication using (medication users) subjects assigned to oral appliance plus mouth shield or oral appliance only.

## **Respiratory dynamics influenced by medication**

Oral appliances reposition the mandible forward and various designs provide similar respiratory event index attenuation responses. (Schneiderman et al. 2020; Ghazal et al. 2009) Whether different oral appliance designs that permit or limit mouth opening have comparable OSA reduction efficacy remains debatable. (Rose et al. 2002) To our knowledge there are no studies comparing paired oral appliance efficacy on OSA reduction addressing possible confounding effects of medication use on respiratory dynamics during sleep.

Study participants not using any prescribed medications and those taking at least one prescribed medication were grouped as oral appliance plus mouth shield or oral appliance only, non-users or medication users, depending upon their random assignment to oral appliance intervention. A list of the study participant’s medication(s) sorted by group are provided in Supplementary Table 1. The reported action the medications used by our study cohort have on the apnea hypopnea index is provided in Table 1.

All individuals allocated to the medication using groups had no co-morbidities that excluded them from the study based on the study’s exclusion criteria. Mann-Whitney *U* tests were used for identifying significant between-group differences for continuous variables. The Student’s *T te*st was used for within group comparison. An adjusted alpha level of 0.0125 was used for each of these pairwise tests; p-values between 0.0125 and 0.05 were characterized as marginally significant. Nonparametric tests were used to determine correlations between variables of interest. To examine the associations with medication users and variables of interest, we calculated odds ratios (OR) and 95% confidence intervals (CI) using logistic regression analysis models adjusting for age and sex. We determined that an OR >1 would indicate a higher probability of medication influence on variables of interest. Conversely, an OR ,<1 would indicate a lower likelihood that medication had influenced the variable of interest.

## **Between group comparison: non-users vs. medication users**

**Respiratory event index (#events/h)**

The respiratory event index at baseline (p=.225) and T2 (p=.164) did not differ between the non-users and medication users. At T3, the respiratory event index was marginally lower in non-users (p=.027) compared with medication users. (Supplementary Table 2) The respiratory event index percent reduction at T2 did not differ between groups. A T3 (p=.028), the respiratory event index percent reduction was marginally greater in the non-medication users compared with medication users. Significant correlations were observed between T3 respiratory event index (p=.013), the respiratory event index percent reduction (p=.025) and medication users.

**Hypopnea index (#events/h)**

The hypopnea index at baseline, T2, and T3 did not differ statistically between non-users and medication users (Supplementary Table 3). At T2, the hypopnea index percent reduction was lower in non-users (p=.007) compared with medication users. This difference was not observed at T3. A positive correlation was observed between T2 hypopnea index percent reduction (p=.006) and medication users.

**Mouth breathing**

No difference in mouth breathing at baseline was observed between non-users and medication users (p=.884). At T3, mouth breathing minutes was lower among non-users (p=.007) compared with medication users (Supplementary Table 4). The mouth breathing percent reduction at T3 from baseline mouth breathing was greater in non-users (p=.001) compared with medication users. Positive correlations were observed between T3 mouth breathing minutes (p=.027), mouth breathing percent reduction (p=.047) and medication users.

**Snore percent**

No differences in snore percent at baseline and T1 - 3 were observed between non-users and medication users. (Supplementary Table 5) The reductions in snore percent at T1 (p=.025) and T3 (p=.038) from baseline were marginally greater in the non-user group compared with medication users. Positive correlations were observed between T1 (p=.023) and T3 (p=.027) snore percent reduction and medication users.

**Average oxygen desaturation (SaO_2_; %) and Oxygen Desaturation Index (ODI; #events/h)**

No significant differences in average SaO_2_ % and ODI were observed between non-users and medication users.

## **Within oral appliance plus mouth shield and oral appliance only group comparison: non-users versus medication users**

**Respiratory rate – supine**

Within oral appliance plus mouth shield and oral appliance only groups, no significant differences in respiratory rate - supine were observed at T1-3 between non-users and medication users.

**Mouth breathing**

Within the oral appliance plus mouth shield group, mouth breathing was marginally lower at T3 compared with baseline among non-users (p=.022) but significantly lower in medication users (p=.007). (Supplementary Table 6). No mouth breathing differences at baseline and T3 were observed between non-users and medication users in the oral appliance plus mouth shield group (p=.668 and p=.234, respectively). However, when one medication user outlier (subject #90; using Tamsulosin, a muscle relaxer) was removed from the analysis, mouth breathing at T3 was marginally higher in the medication users oral appliance plus mouth shield group (p=.014) compared with non-users. The decreased percent change in mouth breathing was marginally greater in non-users (p =.049) compared with medication users. (Supplementary Table 3)

Within the oral appliance only group, no differences in mouth breathing at baseline was observed between non-users and medication users (p=.922). At T3, mouth breathing in non-users and medication users was significantly reduced compared with baseline (non-users, p =.008; medication users (p =.003); respectively). (Supplementary Table 7) At T3, marginally higher mouth breathing was found in medication users (p=.015) users compared with non-users. The mouth breathing percent difference from baseline was significantly greater in non-users (p=.008) compared with mediation users.

**Oxygen Desaturation Index (ODI; #events/h)**

Within the oral appliance plus mouth shield group, the T2 percent ODI reduction from baseline (100%) was greater in non-users compared with medication users (p=.007). (Supplementary Figure 1). No within group percent ODI reduction from baseline differences were observed at T1 and T3.

The oral appliance only group non-users and medication users did not differ significantly in percent ODI reduction from baseline at T1-3.

## **Between oral appliance plus mouth shield and oral appliance only group comparison: non-users versus medication users**

**Respiration rate - supine**

Between oral appliance plus mouth shield and oral appliance only non-users, the respiratory rate - supine change from baseline was decreased at T2 (OA+, -2.7 [-4.5 – -0.8] vs. OA, 0.6[-1.5 – 2.4]; p=.005) with oral appliance plus mouth shield compared with oral appliance only (. No significant differences in the respiratory rate - supine were observed at T1 and T3 in non-users between oral appliance groups.

Medication users respiratory rate – supine was significantly lower at T3 (OA+, -3.4 [-0.8 – -1.1] vs. OA, -0.3[-0.3 – 2.1]; p=.001) with oral appliance plus mouth shield compared with oral appliance only. No significant differences in the respiratory rate - supine were observed at T1 – 2 between medication users in oral appliance plus mouth shield and oral appliance only groups.

**Mouth breathing**

Between groups, comparison of non-users and medication users at baseline showed no significant differences in mouth breathing (p=.884). (Supplementary Table 4) At T3, medication users had significantly higher mouth breathing compared with non-users (p=.007).

## **Non-users versus Antidepressant users**

Sedatives, antidepressants and opioids modulate neuronal circuits that control breathing and the sleep-wake cycle (Robillard R et al. 2021). Adverse effects from these pharmacological groups negatively influence upper airway muscle tone and their effect is three to eight times greater in older adults (Robillard R et al. 2021). Approximately 8.7% of all Americans reported taking antidepressant medication (Edwards et al. 2015).

At sleep onset and during sleep, excitatory serotonergic (5-HT_2A_ receptor) neurotransmission via the hypoglossal nerve (https://www.vet.upenn.edu/*)*, for example, diminishes tongue muscle tone, influences control of upper airway muscle tone and pharyngeal collapsibility (Edwards et al. 2011). A limited number of studies have reported reductions in the apnea hypopnea index following antidepressant intake in people with obstructive sleep apnea (Kraiczi, et al. 1999). Conversely, other reports indicate that antidepressant use may actually exacerbate sleep-disordered breathing (Robillard R et al. 2021).

When antidepressant medication using subjects were removed from our analysis, no significant differences between non-users and the remaining medication using subjects were observed for the variables of interest. This observation was further explored by comparing non-users with antidepressant medication using subjects.

**Respiratory Event Index**

No difference in respiratory event index at baseline was observed between non-users and antidepressant using subjects (p=.273). At T3, respiratory event index was significantly lower in non-users (p=.003) compared with antidepressant users. (Supplementary Table 9) The percent change in the respiratory event index from T3 was marginally greater in non-users (p=.018) compared with antidepressant users. Positive significant correlations were observed between T3 respiratory event index (p=.001), percent respiratory event index difference from baseline (p=.013) and antidepressant users.

**Snore Percent (%)**

No statistical differences between non-users and antidepressant users were observed in the snore percent at baseline, T3, and in T3 percdifference from baseline.

**References**

Brouillette RT, Manoukian JJ, Ducharme FM, Oudjhane K, Earle LG, Ladan S, Morielli A. (2001) Efficacy of fluticasone nasal spray for pediatric obstructive sleep apnea. J Pediatr. 138(6):838-44.

Edwards C, Mukherjee S, Simpson L, Palmer LJ, Almeida OP, Hillman DR. (2015) Depressive Symptoms before and after Treatment of Obstructive Sleep Apnea in Men and Women. J Clin Sleep Med. Sep 15;11(9):1029-38.

Edwards BA, White DP. **(**2011) Control of the pharyngeal musculature during wakefulness and sleep: implications in normal controls and sleep apnea. Head Neck, Suppl 1, 33:S37-45.

Ghazal A, Sorichter S, Jonas I, Rose EC. (2009) A randomized prospective long-term study of two oral appliances for sleep apnoea treatment. J Sleep Res. 18(3):321-8.

https://www.vet.upenn.edu/test-page/old-pages-archive/neuroscience/kubin-laboratory/research-overview/hypoglossal-motoneurons

Kraiczi, H., Hedner, J., Dahlöf, P., Ejnell, H. and Carlson, J. (1999) Effect of serotonin uptake inhibition on breathing during sleep and daytime symptoms in obstructive sleep apnea. *Sleep*, *22*(1), pp.61-67.

Mason M, Cates CJ, Smith I. (2015) Effects of opioid, hypnotic and sedating medications on sleep-disordered breathing in adults with obstructive sleep apnoea. Cochrane Database Syst Rev. 14;(7):CD011090.

Mickelson SA, Lian T, Rosenthal L. (1999) Thyroid testing and thyroid hormone replacement in patients with sleep disordered breathing. ) 78(10):768-71, 774-5.

Moran M. (2016) Reversible exacerbation of obstructive sleep apnea by α1-adrenergic blockade with tamsulosin: A case report. Respir Med Case Rep. 14;19:181-186.

Robillard R, Saad M, Ray RB, Bujaki B, Douglass, Lee EK, et al. (2016) Selective serotonin reuptake inhibitor use is associated with worse sleep-related breathing disturbances in individuals with depressive disorders and sleep complaints: a retrospective study. J Clin Sleep Med. 17(3):505–513.

Rose, E., Staats, R., Virchow, C. and Jonas, I.E. (2002) A comparative study of two mandibular advancement appliances for the treatment of obstructive sleep apnoea. Euro J Orthodontics. 24(2), pp.191-198.

Smales ET, Edwards BA, Deyoung PN, McSharry DG, Wellman A, Velasquez A, et al. (2015) Trazodone Effects on Obstructive Sleep Apnea and Non-REM Arousal Threshold. Ann Am Thorac Soc. 12(5):758-64.

Schneiderman E, Schramm P, Hui J, Wilson PD, Moura P, German Z, et al. (2021*)* Randomized Trial of 2 Self-Titrated Oral Appliances for Airway Management. J Dent Res. Feb;100(2):155-162.

Tamura A, Kawano Y, Naono S, Kotoku M, Kadota J. (2007) Relationship between beta-blocker treatment and the severity of central sleep apnea in chronic heart failure. Chest. 131(1):130-5.

### Supplementary Table 1. List of prescribed medications taken by oral appliance plus mouth shield and oral appliance only group and their impact on the apnea hypopnea index.

| Medication | Group | | Drug Class | Action | Ref. |
| --- | --- | --- | --- | --- | --- |
|  | OA+ | OA |  |  |  |
| Bupropion;Venlafaxine |  | 2 | SNRI | Increases AHI in depression | (Robillard R et al. 2021) |
| Levothyroxine | 2 | 1 | Synthetic hormone | T4 replacement does not improve OSA | (Mickelson SA et al. 1999) |
| Escitalopram |  | 1 | SSRI | Increases AHI in depression | (Robillard R et al. 2021) |
| Fluoxetine; Methylphenidate |  | 1 | SSRI; Phenethylamines | Increases AHI in depression | (Robillard R et al. 2021) |
| Zolpidem (+Benzodiazepine – OA+) | 1 | 1 | Sedatives and hypnotics; ‘Z drugs’; Non-benzodiazepine; (GABA receptor agonist) | Non-significant increase in AHI; significant SaO_2_ desaturation increase with 20mg | (Mickelson SA et al. 1999) |
| Propranolol |  | 1 | β1-blocker | Lower AHI in CHF patients | (Tamura A et al. 2007) |
| Trazodone |  | 1 | SARI | Non-significant AHI increase in NREM sleep stages 2 and 3, but reduced AHI in Stage 1 | (Smales ET et al. 2015) |
| Sertraline | 1 | 1 | SSRI | Increases AHI in depression | (Robillard R et al. 2021) |
| Fluticasone | 2 | 1 | Trifluorinated glucocorticoid (intranasal) | Decreased AHI in children | (Brouillette RT et al. 2001) |
| Tamsulosin | 1 |  | α1-blocker; Anti-muscarinic | Increases AHI (on CPAP) | (Moran M. et al. 2016) |

Abbreviations: AHI, apnea hypopnea index; SNRI, serotonin norepinephrine reuptake inhibitor, SSRI, selective serotonin reuptake inhibitor; GABA, gamma-aminobutyric acid; SARI, serotonin antagonist reuptake inhibitor; CHF, congestive heart failure; CPAP, continuous positive airway pressure; OSA, obstructive sleep apnea; OA+, oral appliance plus mouth shield, OA, oral appliance only.

### Supplementary Table 2. Medication use status on respiratory event index (#events/h)

| Variable | Non-users | Medication users | P value | Spearman’s rho Corr. Coefficient | P value |
| --- | --- | --- | --- | --- | --- |
| Baseline respiratory event index | 25.1[15.1 – 45.0] | 18.4[14.2 – 27.8] | .225 | .205 | .231 |
| T2 respiratory event index | 7.3[4.5 – 14.6] | 4.3[2.7 – 11.4] | .164 | -.178 | .166 |
| T3 respiratory event index | 6.4[3.1 – 7.9] | 17.8[9.7– 25.7] | .027 | .372 | .013 |
| T2 Respiratory event index % reduction from Baseline | -63.6[-86.0 – -23.6] | -71.9[-80.9 – -48.9] | .544 | -.106 | .552 |
| T3 Respiratory event index % reduction from Baseline | -71.5[-87.4 – -53.5] | -46.7[-68.0 – 26.2] | .028 | .408 | .025 |

Abbreviations: non-users of prescribed medication (OA+, n=7; OA only, n=6); medication users (OA+, n=6; OA only, n=11); T3, 8-weeks intervention use.

### Supplementary Table 3. Medication use status on hypopnea index (#events/h)

| Variable | Non-users | Medication users | P value | Spearman’s rho Corr. Coefficient | P value |
| --- | --- | --- | --- | --- | --- |
| Baseline hypopnea index | 14.3[10.6 – 17.9] | 11.6[7.2 – 19.4] | .124 | -.260 | .126 |
| T2 hypopnea index | 4.0[2.1 – 6.9] | 6.8[3.1 – 15.5] | .215 | .160 | .218 |
| T3 hypopnea index | 4.4[2.7 – 11.3] | 6.0[2.5 – 13.6] | .095 | .233 | .096 |
| T2 Hypopnea index % reduction from Baseline | -65.6[-83.7 – -49.6] | -28.0[-61.6 – 17.9] | .007 | .348 | .006 |
| T3 Hypopnea index % reduction from Baseline | -55.6[-79.5 – -34.8] | -51.0[-68.1 – -33.0] | .331 | .239 | .088 |

Abbreviations: non-users – no prescribed medication (OA+, n=7; OA only, n=6); Medication users – prescribed medication (OA+, n=6; OA only, n=11); T3, at 8-weeks intervention use; OA+, oral appliance plus mouth shield, OA, oral appliance only.

### Supplementary Table 4. Medication use status on mouth breathing

| Variable | Non-users | Medication users | P value | Spearman’s rho Corr. Coefficient | P value |
| --- | --- | --- | --- | --- | --- |
| Baseline mouth breathing (minutes) | 128.1[62.3 – 150.1] | 115.1[70.3 – 147.2] | .884 | .016 | .935 |
| T3 mouth breathing (minutes) | 0.0[-0.58 – 10.8] | 12.7[7.1 – 43.2] | .007 | .404 | .027 |
| T3 Mouth breathing % reduction from baseline | -100.0[-100.4 – -93.2] | -80.3[-87.5 – -67.0] | .001 | .365 | .047 |

Abbreviations: non-users – no prescribed medication (OA+, n=7; OA, n=6); medication users – prescribed medication (OA+, n=6; OA, n=11); T3, at 8-weeks intervention use; OA+, oral appliance plus mouth shield, OA, oral appliance only.

### Supplementary Table 5. Medication use status on snore percent

| Variable | Non-users | Medication users | P value | Spearman’s rho Corr. Coefficient | P value |
| --- | --- | --- | --- | --- | --- |
| Baseline snore percent (%) | 28.2 [5.6 – 39.0] | 19.2 [9.6 – 26.7] | .265 | .016 | .935 |
| T1 snore percent (%) change from Baseline | -22.1 [-33.8 – 2.8] | -6.5 [-16.5 – 9.6] | .025 | .295 | .023 |
| T2 snore percent (%) change from Baseline | -16.4 [-28.6 – 4.4] | -10.5 [-18.9 – 0.1] | .209 | .161 | .212 |
| T3 snore percent (%) change from Baseline | -11.0 [-31.7 – 4.1] | -4.8 [-14.8 – 9.7] | .038 | .293 | .037 |

Abbreviations: non-users – no prescribed medication (OA+, n=7; OA only, n=6); medication users – prescribed medication (OA+, n=6; OA only, n=11); T3, at 8-weeks intervention use; OA+, oral appliance plus mouth shield, OA, oral appliance only.

### Supplementary Table 6. Comparison of mouth breathing within oral appliance plus mouth shield group: non-users versus medication users

|  | OA+ Group non-users versus medication users (median [IQR]) | | | | P value | | | |
| --- | --- | --- | --- | --- | --- | --- | --- | --- |
|  |  |  |  |  | **Non-users** | **Medication users** | **Baseline** | **T3** |
|  | **Non-users baseline** | **Medication users baseline** | **Non-users_T3** | **Medication users_T3** | **Baseline vs. T3** | **Baseline vs. T3** | **Non-users vs. Medication users** | **Non-users vs. Medication users** |
| T3 Mouth breathing (minutes) | 134.05  [42.23 – 158.23] | 120.60  [57.85 – 142.00] | 0.00  [0.00 –- 11.78] | 3.40  [1.05 – 37.05] | .022 | .007 | .668 | .234* |
| T3 Mouth breathing % reduction from baseline |  |  | -100.00  [-100.00 – -93.75] | -78.49  [-90.19 – -63.67] |  |  |  | .049 |

*p=.014 when outlier (using Tamsulosin) is removed; Abbreviations: OA+ non-users – no prescribed medication (n=6); OA+ medication users – prescribed medication (n=7); IQR, Interquartile range; OA+, oral appliance plus mouth shield.

### Supplementary Table 7. Mouth breathing comparison within oral appliance only group: non-users versus medication users

|  | Oral appliance only group:  non-users versus medication users (median [IQR]) | | | | P value | | | |
| --- | --- | --- | --- | --- | --- | --- | --- | --- |
|  |  |  |  |  | **Non-users** | **Medication users** | **Baseline** | **T3** |
|  | **Non-users baseline** | **Medication users baseline** | **Non-users_T3** | **Medication users_T3** | **Baseline vs. T3** | **Baseline vs. T3** | **Non-users vs. Medication users** | **Non-users vs. Medication users** |
| Mouth breathing (minutes) | 83.10  [49.95 – 172.60] | 103.60  [48.30 - 172.20] | 0.00  [0.00 - 1.40] | 14.90  [5.40 – 33.13] | .008 | .003 | .922 | .015 |
| T3 Mouth breathing % reduction from baseline |  |  | -100.00  [-100.00 - -99.29] | -85.21  [-90.63 - -73.76] |  |  |  | .008 |

Oral appliance only non-users – no prescribed medication (n=7); Oral appliance only medication users – prescribed medication (n=10); IQR, Interquartile range;

### Supplementary Table 8. Mouth breathing comparison between: non-users versus antidepressant users

|  | Non-users versus Medication users (median [IQR]) | | | | | | | | | | P value | | | | | |
| --- | --- | --- | --- | --- | --- | --- | --- | --- | --- | --- | --- | --- | --- | --- | --- | --- |
|  |  | | | | | | | | | | **Non-users  OA+ vs. OA** | | | | **Medication users OA+ vs. OA** | |
|  | **Non-users Baseline** | | **Non-users T3** | | | **Medication users Baseline** | | | **Medication users T3** | | **Base line** | | **T3** | | **Baseline** | **T3** |
|  | **OA+** | **OA** | **OA+** | **OA** | **OA+** | | **OA** | **OA+** | | **OA** | |  | |  |  |  |
| Mouth breathing (minutes) | 134.05  [42.23 – 158.23] | 83.10  [49.95 – 172.60] | 0.00  [0.00 – 11.78] | 0.00  [0.00 – 1.40] | 120.60  [57.85 – 142.00] | | 103.60  [48.30 – 172.20] | 0.00  [0.00 – 1.40] | | 14.90  [5.40 – 33.13] | | .668 | | .861 | .922 | .493 |
| T3 Mouth breathing % difference from baseline |  |  | -100.00  [-100.00 – -93.75] | -100.00  [-100.00 - -99.29] |  | |  | -78.49  [-90.19 – -63.67] | | -85.21  [-90.63 – -73.76] | |  | | .727 |  | .696 |

Abbreviations: OA+ non-users – no prescribed medication (n=6); OA+ medication users – prescribed medication (n=7); OA non-users – no prescribed medication (n=7); OA medication users – prescribed medication (n=10); IQR, Interquartile range;

### Supplementary Table 9. Respiratory event index comparison between: non-users versus antidepressant users

| Variable | Non-users | Antidepressant users | P value | Spearman’s rho Corr. Coefficient | P value |
| --- | --- | --- | --- | --- | --- |
| Baseline respiratory event index | 25.1[15.1 – 45.0] | 18.4[14.2 – 27.8] | .273 | -.259 | .285 |
| T3 respiratory event index | 6.4[3.1 – 7.9] | 17.8[9.7– 25.7] | .003 | .703 | .001 |
| T3 respiratory event index reduction from Baseline | -16.1[-35.6 – -10.9] | -4.3[-12.3 –-6.3] | .018 | .558 | .013 |

Abbreviations: Non-users – no prescribed medication (n=13); antidepressant users- prescribed antidepressant medication (n=6); T3, 8-weeks intervention use.
